# Ectopic Expression of *OsSta2* Enhances Salt Stress Tolerance in Rice

**DOI:** 10.3389/fpls.2017.00316

**Published:** 2017-03-10

**Authors:** Manu Kumar, Juyoung Choi, Gynheung An, Seong-Ryong Kim

**Affiliations:** ^1^Department of Life Science, Sogang UniversitySeoul, South Korea; ^2^Department of Plant Molecular Systems Biotechnology, Kyung Hee UniversityYongin, South Korea

**Keywords:** abiotic stress, salt stress, drought stress, osmotic stress, rice, ABA, agronomic trait, tiller number

## Abstract

Salt stress can severely reduce crop yields. To understand how rice (*Oryza sativa*) plants respond to this environmental challenge, we investigated the genes involved in conferring salt tolerance by screening T-DNA tagging lines and identified *OsSta2-D* (*Oryza sativa S*alt *t*olerance *a*ctivation *2*-*D*ominant). In that line, expression of *OsSta2* was enhanced by approximately eightfold when compared with the non-transformed wild type (WT). This gene was highly expressed in the callus, roots, and panicles. To confirm its role in stress tolerance, we generated transgenic rice that over-expresses *OsSta2* under a maize *ubiquitin* promoter. The *OsSta2-*Ox plants were salt-tolerant at the vegetative stage, based on our calculations of chlorophyll fluorescence (Fv/Fm), fresh and dry weights, chlorophyll concentrations, and survival rates. Under normal paddy field conditions, the Ox plants were somewhat shorter than the WT control but had improved agronomic traits such as higher total grain yield. They were also more tolerant to osmotic stress and hypersensitive to abscisic acid. Based on all of these results, we suggest that *OsSta2* has important roles in determining yields as well as in conferring tolerance to salt stresses.

## Introduction

For more than half of the world’s people, rice (*Oryza sativa*) is a major food crop. Global demand for this grain will rise as populations continue to grow. Diverse environmental stresses cause plants to respond at the molecular level by altering the expression of different sets of regulatory or signaling genes as well as genes that encode proteins related to stress tolerance (Apse and Blumwald, 2002; Seki et al., 2003; Shinozaki et al., 2003; Wang et al., 2003; Kumar et al., 2013; Fahad et al., 2015; Kazan, 2015; Parihar et al., 2015; Petrov et al., 2015). Drought and salt stresses are common environmental factors that restrict rice productivity (Yeo and Flowers, 1984; Xoconostle-Cázares et al., 2010; Das et al., 2015; Fita et al., 2015). On high-salinity soils, annual grain yields can be reduced by 30–50% (Eynard et al., 2005). Significant progress has been made in understanding the mechanism(s) for salt tolerance in many plant species, including rice (Kumar et al., 2013; Deinlein et al., 2014; Parihar et al., 2015). Under salt stress, cells can be protected and normal plant growth maintained through cellular responses such as cytosolic calcium release, ionic imbalances in the vacuole, stress signal transduction, and expression of several regulatory genes (Kasuga et al., 1999; Kader and Lindberg, 2010; Ismail et al., 2014). Because all of these responses indicate that various species utilize a common set of signaling pathways and genes, researchers can exploit this to engineer plants with greater salt tolerance.

Transcription factors (TFs) such as AP2/ERF, bZIP, MYB, NAC, zinc-finger, MYC, and WRKY are important because they can regulate the downstream expression of many stress-responsive genes (Bhatnagar-Mathur et al., 2008; Joshi et al., 2016; Wang et al., 2016). Transgenic application of TFs is a useful approach for developing plants that are more tolerant to abiotic stresses. Among them, AP2/ERFs have multiple roles in plants, controlling processes such as, leaf epidermal cell identity; the development of leaf petioles, flowers, and embryos; and fruit ripening (Elliott et al., 1996; Moose and Sisco, 1996; van der Graaff et al., 2000; Boutilier et al., 2002; Wang et al., 2007; Krizek, 2009; Licausi et al., 2013).

The AP2/ERF proteins are also involved in plant responses to biotic stress. For example, ERF proteins modulate the expression of many pathogenesis-related genes by binding to GCC box (AGCCGCC) (Ohme-Takagi and Shinshi, 1995; Solano et al., 1998; Fujimoto et al., 2000; Gu et al., 2002; Onate-Sanchez and Singh, 2002; Onate-Sanchez et al., 2007; Liu et al., 2012; Zhao et al., 2012; Jisha et al., 2015; Mishra et al., 2015). Proteins such as ERN1, -2, -3, and EFD from *Medicago truncatula* regulate the development of legume root nodules to establish symbiosis with nitrogen-fixing bacteria (Andriankaja et al., 2007; Middleton et al., 2007). Likewise, the miR172-AP2-1 node acts as a key regulator of nitrogen fixation in the symbiotic relationship of *Phaseolus vulgaris*–*Rhizobium etli* (Nova-Franco et al., 2015).

Apart from their role in biotic stress responses, AP2/ERF proteins also participate in response to abiotic stresses such as drought, salt, and cold (Nakano et al., 2006; Xu et al., 2011; Mizoi et al., 2012; Licausi et al., 2013; Fu et al., 2014; Jisha et al., 2015). These proteins contain a conserved AP2/ERF domain (Riechmann et al., 2000; Sharoni et al., 2011; Licausi et al., 2013). One of the best-studied is a group of CBF/DREBs that activate the expression of many stress-related genes and improve drought, salt, and cold tolerance (Stockinger et al., 1997; Liu et al., 1998; Kasuga et al., 1999; Sakuma et al., 2006; Lata and Prasad, 2011; Schmidt et al., 2013; Zhuang et al., 2013; Rong et al., 2014; Yang et al., 2014, 2016; Duan et al., 2015).

Rice (*O*. *sativa* ssp. *japonica*) has at least 139 AP2/ERF family genes (Nakano et al., 2006), and various environmental stresses induce their expression (Dubouzet et al., 2003; Cao et al., 2006; Fukao et al., 2006; Xu et al., 2006; Liu et al., 2007; Hattori et al., 2009). For example, genes for the AP2/ERF proteins SNORKEL1 and SNORKEL2 promote the accumulation of gibberellic acid in deep-water rice and rapid stem elongation under flooding conditions as an escape strategy (Hattori et al., 2009). In contrast, the AP2/ERF protein SUB1A-1 in submergence-tolerant rice varieties is part of a quiescence strategy that prevents shoot elongation and increases their rate of survival (Xu et al., 2006). Constitutive expression in rice of AP2/ERF genes such as *DREB1A, HARDY* (from *Arabidopsis*), *HvCBF4* (from *Hordeum vulgare*), and *TERF1* (from *Solanum lycopersicon*) enhances tolerance to abiotic stress (Oh et al., 2005, 2007; Karaba et al., 2007; Gao et al., 2008), while overexpression of the rice AP2/ERF gene *AP37* increases drought tolerance at the vegetative stage and leads to higher grain yields (Oh et al., 2009). Overexpression in rice of *TSRF1*, another AP2/ERF protein, also improves tolerance to osmotic stress and drought (Zhang et al., 2004, 2007; Quan et al., 2010). Salt-responsive ERF1 regulates reactive oxygen species-dependent signaling during the initial response to salt stress in rice (Schmidt et al., 2013) while the rice ERF TF factor *OsERF922* negatively regulates resistance to the development of salt tolerance (Liu et al., 2012). Furthermore, overexpression of rice *OsEREBP1* increases tolerance to both biotic and abiotic stresses (Jisha et al., 2015). Based on these earlier reports, rice functional genomics, including reverse and forward genetics methods, is now an important research field for identifying novel genes involved in plant stress responses and tolerance. These genes can become new targets for genetic engineering of rice and other crops to improve tolerance.

In this study, we characterized a gene that is induced by several types of stress. Overexpression of *OsSta2* made rice plants more tolerant to oxidative and salt stresses at the seedling and vegetative stages, respectively. This overexpression also helped improve overall agronomical traits under normal paddy field conditions.

## Materials and Methods

### Plant Materials

Rice (*O. sativa* ssp. *japonica* cv. Dongjin) seeds were surface-sterilized and germinated in a wet paper towel for 2 days. The resultant seedlings were cultured in a walk-in growth chamber (Koencon, South Korea) under conditions of 30°C [day/22°C (night) and a 12-h photoperiod (Lee et al., 2015)].

### Abiotic Stress Treatments and Assays of Stress Tolerance

Gene expression was analyzed using rice seedlings that had been hydroponically cultured in Yoshida solution (Yoshida et al., 1976). At 8 DAG, they were exposed to various types of stress for 0, 1, 3, 6, 12, or 24 h. The treatments included drought (water removal), salt (300 mM NaCl), cold (4°C), or abscisic acid (100 μM ABA). After the treatment period, 100 mg leaf tissue was collected for RNA extraction.

To test the extent of tolerance in our transgenic rice lines, we sowed seeds in a soil box. At 8 DAG, the seedlings underwent drought stress when water was withheld for 30–40 h until the leaves wilted. To induce salt stress, 8 DAG seedlings were transferred to either 100 mM NaCl for 7 days or 250 mM NaCl solution for 72 h. To examine their response to a low temperature, we incubated 8 DAG seedlings for 48–72 h at 4°C (Koencon, South Korea). At the end of each treatment period, the plants were returned to normal growing conditions for 6 days of recovery before their phenotypes were recorded and their survival rates were calculated. For all treatments, dry weights were determined after the plants had been dried at 80°C for 2 days.

To examine osmotic stress tolerance and ABA sensitivity, we germinated surface-sterilized, de-hulled rice seeds on a half-strength MS medium for 5 days before transferring the seedlings to a half-strength MS medium supplemented with 0 or 200 mM mannitol, or with 0, 5, or 10 μM ABA. Seedlings were oriented vertically and their growth was observed 7 days after this transfer (Kim H. et al., 2012). The stress tolerance assay also included an examination of chlorophyll fluorescence. Briefly, the fifth leaves from 12 DAG seedlings were removed and incubated in 500 mM NaCl for 48 h, then either air-dried for 3 h (28°C; 110 μmol m^-2^ s^-1^) or incubated at 4°C in deionized water for up to 48 h (4°C; 110 μmol m^-2^ s^-1^). The *Fv*/*Fm* values, which represent the photochemical efficiency of PSII in a dark-adapted state (*Fv*, variable fluorescence; *Fm*, maximum fluorescence) were calculated with data obtained by using a Mini-PAM-II Photosynthesis Yield Analyzer (Walz, Germany). A leaf disk assay was conducted to examine salt tolerance. Healthy and fully expanded leaves (∼60 DAG) were washed in deionized autoclaved water before 1-cm-diameter disks were cut and floated for 24 h in 30-mL solutions of various concentrations of NaCl (100, 200, or 250 mM) (Tuteja et al., 2013). The effects of salt stress were represented as phenotypic changes and quantifications of chlorophyll (Arnon, 1949). Briefly, 1-cm disks were ground and extracted with 80% acetone. Absorption was measured at 645 and 663 nm with a spectrophotometer (Shimadzu, Japan).

### Screening of Activation Tagging Lines for Salt Tolerance

Rice T-DNA tagging mutants were screened for salt tolerance (100 or 250 mM NaCl) by using a mixed pool of approximately 5,000 individuals from the T2 generation of a T-DNA ATL (Jeong et al., 2002, 2006). After 2–7 days of induced salt stress, followed by 6 days of recovery, a mutant line showing enhanced tolerance (based on a high survival rate) was identified and further characterized.

Inverse PCR (IPCR) was performed by *Cla*1 cutting in our pGA2715-tagged lines (Jeong et al., 2002; Jung et al., 2003), the primers for the 1st and 2nd IPCR included in Supplementary Table S2. Samples were amplified for 35 cycles of 94°C for 1 min, 58°C for 1 min, and 72°C for 5 min. Aliquots from the primary PCR products were used for the secondary PCR reaction and then the PCR products were directly sequenced. Genomic sequences containing the tagging sequence were retrieved from Rice GE Database^1^.

### Gene Expression Analysis by RT-PCR and qRT-PCR

Total RNA was isolated from rice leaf samples with an RNeasy Mini Plant Kit (Qiagen, Germany) and cDNAs were synthesized with RT Complete Kits (Biofact, South Korea), according to the manufacturers’ instructions. Primers were designed with Gene Runner software^2^ and NCBI primer blast^3^. Primer pairs (Supplementary Table S1) were used at concentration of 5–10 picomoles. In addition, 3 μL of cDNA (6 ng of total RNA) was used as template. All RT-PCRs were performed at an initial 95°C for 5 min, followed by 25–35 cycles of 95°C for 30 s, 58°C for 30–60 s, and 72°C for 30–60 s. The PCR products were visualized on a 0.8% agarose gel. The qRT-PCR analysis utilized a SYBER^®^ FAST Universal qPCR Kit (Kapa, South Africa) and a LightCycler^®^ 96 (Roche Life Science, Germany). The qPCR procedures were performed at 95°C for 3 min, followed by 40 amplification cycles of 95°C for 3 s, 60°C for 20 s, and 72°C for 20 s. A melting curve was obtained through a protocol involving 95°C for 5 s, 65°C for 1 min, and 97°C for 1 min; followed by cooling at 40°C for 10 min. Relative expression levels were calculated by the 2^-ΔΔCt^ method (Livak and Schmittgen, 2001), using *RAc1* as an internal control.

### *In silico* Analysis of the *OsSta2* Promoter

Promoter sequences (approximately 2 kb long) upstream of the ATG start codon were analyzed from Oryzabase (Kurata and Yamazaki, 2006), and *cis*-elements in those promoters were searched in the PLACE database^4^ (Higo et al., 1999).

### Generation of *OsSta2* Overexpression Lines

For construction of the *OsSta2*-overexpression (-Ox) vector, *OsSta2* cDNA (J065129D08) was obtained from KOME^5^. The cDNA was placed between the *Sac*I and *Bam*HI sites by subcloning and then cloned in to the pGA3426 binary vector with a maize *ubiquitin* promoter and the *nos* terminator (Kim et al., 2009). Scutellum-derived calli of ‘Dongjin’ rice were transformed by *Agrobacterium*-mediated co-cultivation methods. 5 days scutella were used for transformation experiments. Subculture was done for 4 days in 2N6 medium (Hiei et al., 1994; Koh et al., 2007). The transgenic plants were then transferred to a confined paddy field for further growth. For segregation analysis of the transgenic lines, seeds were germinated in a half-strength MS medium supplemented with hygromycin (50 mg L^-1^). The number of surviving seedlings was recorded after incubation at 30°C for 5 days. Lines in which the survival rate was 100% were considered transgene homozygotes.

### Investigation of Agricultural Traits

Rice plants were grown from May until the grain was harvested at the end of October. These experiments were conducted annually for 4 years, 2012 through 2015, at the LMO paddy field of Kyungpook National University and Kyung Hee University, South Korea (Permit number, RDA-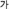
A-2011-039). To analyze the agricultural traits of rice, we sampled eight plants from each of three independent lines and recorded the numbers of tillers and panicles per plant, the numbers of spikelets and filled grains per panicle, lengths of the panicles and culms, and 1,000-grain weights.

### Statistical Analysis

Mean values (±SE) were determined from the data set for three replications. Differences between stress treatments were examined with LSD and χ^2^ tests, and were considered statistically significant at *P* < 0.05.

## Results

### Isolation of a Salt Stress-Tolerant Activation Tagging Line

**FIGURE 1:**
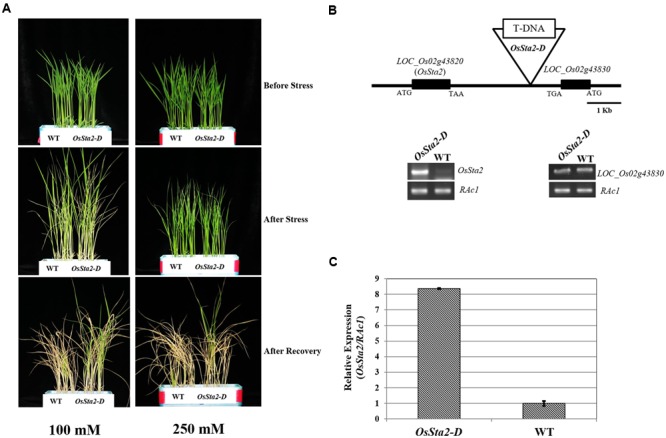
**Identification of salt-tolerant activation tagging line (ATL) *OsSta2-D*. (A)** Visual phenotypes of salt response by ATL and WT after 8 DAG seedlings were exposed to either 100 mM NaCl for 7 days or 250 mM NaCl for 48 h. All seedlings were returned to normal conditions for 6 days of recovery. **(B)** Schematic diagram of ATL of *OsSta2* and RT-PCR of *LOC_Os02g43830* (*OsSta2*) and *LOC_Os02g43830*. **(C)** qRT-PCR of ATL showing *OsSta2* mRNA levels isolated from young seedlings. Error bars represent standard error of three replicates.

Screening a mixed pool of the T2 generation of PFG T-DNA tagging mutants (Lee et al., 2004; Jeong et al., 2006), we identified Line PFG_3A-05272.R, which had enhanced tolerance to treatments with 100 mM and 250 mM NaCl (**Figure 1A** and **Table 1**). Molecular analysis by inverse PCR revealed that the T-DNA was tagged between *LOC_Os02g43820* and *LOC_Os02g43830* (**Figure 1B**). However, expression of only *LOC_Os02g43820* was induced, by eightfold, when compared with the wild type (WT) (**Figure 1C**). This gene was named *Oryza sativa S*alt *t*olerance *a*ctivation *2*-*D*ominant, or *OsSta2-D* (*AK241246*). Its deduced amino acids contain a 775-bp open reading frame that yields a 56-amino acid protein. Potential stress-related *cis*-acting elements like, W-box, GT1, MYB, MYC, GATA box, ABRE and ERd1, etc., were found in the 2 kb upstream region of *OsSta2* (Buchel et al., 1999; Finkelstein and Lynch, 2000; Chen et al., 2002; Xue, 2002; Yang and Poovaiah, 2002; Itzhaki et al., 1994; Xie et al., 2005) (Supplementary Figure S1 and Table S1).

**Table 1:** Level of salt tolerance in WT rice and *OsSta2-D* lines, based on survival rates of 8 DAG seedlings exposed to salinity treatment (100 mM NaCl for 7 days or 250 mM NaCl for 48 h) and then returned to normal growing conditions for 6 days of recovery.

Treatment	WT	*OsSta2-D*
100 mM NaCl	9/48^a^ (18.8)^b^	16/43^∗^ (37.2)
250 mM NaCl	0/48 (0.0)	5/48^∗^ (10.4)

*^a^Number of surviving seedlings/total number of seedlings tested; ^b^Percent survival; ^∗^values are significantly different from those of WT at *P* < 0.05.*

### Expression Analysis of *OsSta2*

**FIGURE 2:**
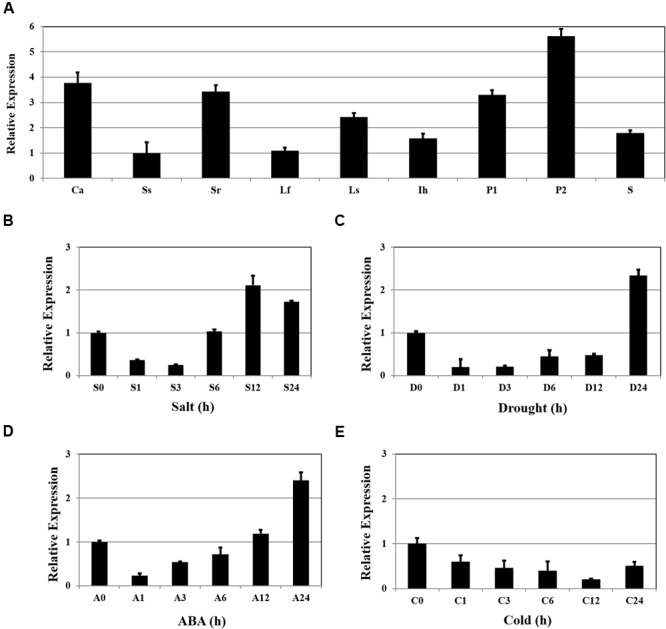
**Patterns of *OsSta2* expression under stress conditions and in developing tissues. (A)** qRT-PCR analysis of *OsSta2* throughout various stages of development. Ca, callus; Ss, 7 DAG shoot; Sr, 7-day-old root; Lf, mature leaf; Ls, flag leaf sheaths; Ih, highest internodes at pre-heading stage; P1, 3- to 8-cm-long panicles; P2, mature panicles; S, mature seed at 3 days after pollination (DAP). *RAc1* was used as reference gene. qRT-PCR analysis of *OsSta2* detected in stress-treated seedlings after 0, 1, 3, 6, or 24 h of exposure to salt **(B)**, drought **(C)**, ABA **(D)**, or cold **(E)**. Error bars represent standard error of three replicates.

Expression of *OsSta2* was examined by RT-PCR and validated by qRT-PCR. Although the gene was detected in all tissue types, transcripts were more abundant in the panicles, callus, and 7 DAG roots, while levels were relatively low in 7 DAG shoots (**Figure 2A**). Expression increased by approximately twofold after 12 h of salt stress (**Figure 2B**), and after 24 h of drought or ABA treatment (**Figures 2C,D**), but was not induced under cold stress (**Figure 2E**).

### Generation of *OsSta2* Transgenic Rice

**FIGURE 3:**
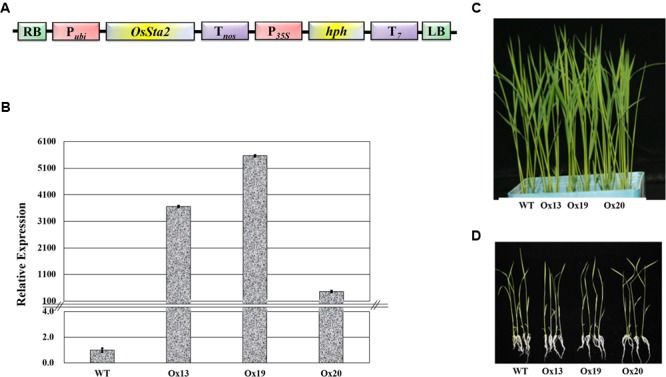
**Production of transgenic rice over-expressing *OsSta2.* (A)** Schematic diagram of Pubi::*OsSta2* construct used for transformation; P*35S*, CaMV 35S promoter; P*ubi*, maize *ubiquitin* promoter; T*7*, terminator sequence of Transcript *7*; T*nos*, terminator sequence of *nopaline synthase*; *hph, hygromycin phosphotransferase*; RB and LB, right and left border sequences of Ti plasmid from *A*. *tumefaciens.* **(B)** qRT-PCR analysis of *OsSta2* in transgenic overexpression (Ox) rice lines. Actin was loading control. **(C)** Visual phenotype for 8 DAG seedlings from *OsSta2*-Ox line grown in soil box. **(D)** Visual phenotype of 14 DAG seedlings grown in half-strength MS medium.

A full-length cDNA sequence (J065129D08) obtained from KOME was incorporated under the maize *ubiquitin* promoter in the pGA3426 vector (**Figure 3A**). pGA3426 vector has T7 terminator in T-DNA which have been used for expression of foreign gene (Jeon et al., 2000). We could not clone the full-length cDNA as reported by Fu et al. (2007), and could not even detect any transcript spanning the putative AP2 domain (Supplementary Table S2 and Figure S4). The cassette was transformed into ‘Dongjin’ rice and 21 independent transgenic lines were generated. The insertion of *OsSta2* was confirmed by PCR analyses of the genomic DNA (Supplementary Figure S2). From those primary transgenic plants, we chose five lines with normal seed formation and used them for T1 production in the confined paddy field. Seeds were harvested and subjected to selection on a hygromycin-containing medium for segregation analysis. Three T2 overexpression lines (Ox13, Ox19, and Ox20) that over-expressed *OsSta2* were identified by RT-PCR and validated by qRT-PCR analysis (**Figure 3B**). Different generations of overexpression lines were used for different set of experiments (Supplementary Figure S5). Southern blot analysis was done to check the copy number integration in three independent overexpression lines by digesting 4 μg DNA with *Hind*III, *EcoR*I and *BamH*I restriction enzyme (Supplementary Figure S6). None of those independent lines differed morphologically from each other when grown under normal conditions either in a soil box or on half-strength MS media (**Figures 3C,D**).

### Overexpression of *OsSta2* in Transgenic Rice Plants Enhances their Salt Tolerance at the Vegetative Stage

**FIGURE 4:**
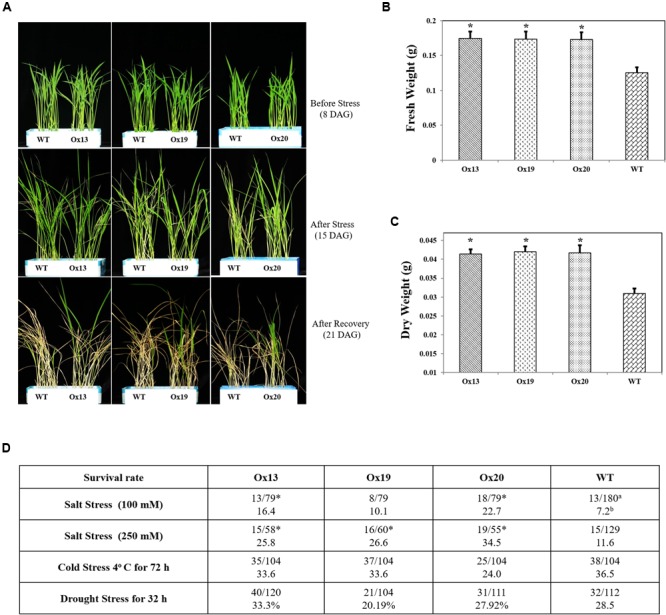
**Enhanced salt tolerance in transgenic rice over-expressing *OsSta2* at vegetative stage. (A)** Visual phenotype of salt response by Ox transgenic plants and WT after 8 DAG seedlings were exposed to 100 mM NaCl for 7 days before returning to normal conditions for 6 days of recovery. **(B)** Fresh weights after recovery. **(C)** Weights after plants were dried for 2 days. **(D)** Stress tolerance of *OsSta2-*Ox lines based on 8 DAG seedlings treated with drought (water withheld for 32 h), salt (100 mM NaCl treatment for 7 days or 250 mM NaCl treatment for 72 h), or cold (4°C for 72 h). Percent survival was calculated after recovery period. ^a^Number of surviving seedlings/total number of seedlings tested; ^b^Percent survival; ^∗^, results are significantly different between Ox line and WT at *P* < 0.05.

To investigate whether overexpression of *OsSta2* can confer salt tolerance at the vegetative stage, we exposed rice seedlings to 100 mM NaCl for 7 days and found that 10.1–22.7% of the *OsSta2*-Ox transgenics survived versus 7.2% of the WT plants. Similar results were obtained after treatment with 250 mM NaCl for 72 h, with 25.8–34.5% survival for the transgenics versus 11.6% survival for the WT (**Figures 4A–C**). After the recovery period, fresh and dry weights were 2–11% higher for the *OsSta2*-Ox plants than for the WT (**Figure 4** and Supplementary Figure S3). Drought tolerance was not improved in the transgenics at the vegetative stage.

**FIGURE 5:**
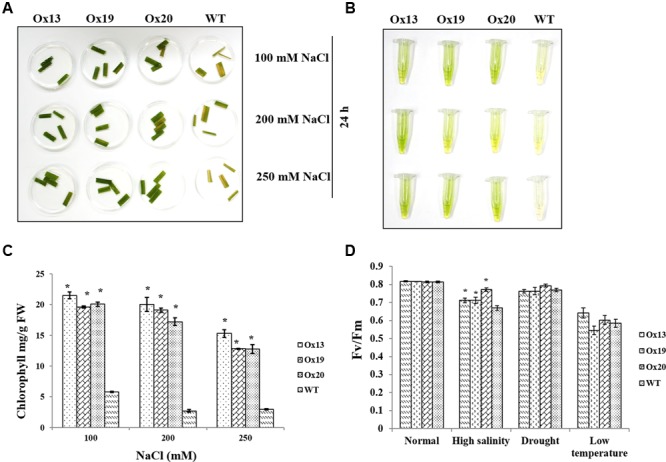
**Comparison of photosynthetic rates under stress conditions. (A)** Leaf disk assays of homozygous T4 *OsSta2*-Ox plants, WT, and ATL under salinity stress (100, 200, or 250 mM NaCl) after 24 h. **(B,C)** Estimates of chlorophyll concentration (mg per g fresh weight) in homozygous T4 *OsSta2*. **(D)** Changes in chlorophyll fluorescence (*Fv*/*Fm*) in leaves sampled from rice plants grown in soil for 12 days before being exposed to high salinity (500 mM NaCl for 48 h), drought (lack of water in Petri plate for 3 h), or low-temperature stress (4°C for 48 h) under continuous light (150 μmol m^-2^ s^-1^). Three independent homozygous T4 lines of *OsSta2*-Ox plants and WT controls were tested. ^∗^, differences between Ox line and WT are significant at *P* < 0.05.

Leaf disk assays performed under various concentrations of NaCl revealed that less chlorophyll was lost from the Ox lines than from the WT plants (**Figures 5A,B**). For example, in response to 100 mM NaCl, the WT samples contained 5.8 mg of chlorophyll per g of leaf tissue versus 19.6–21.5 mg per g in the transgenics, i.e., 14–16% more than in the WT. Similar results were obtained in response to 200 or 250 mM NaCl. There, chlorophyll concentrations in the WT ranged from 3.0 to 6.2 mg per g, which was 9–12% lower than the 12.0–20.0 mg g measured in the Ox lines. These higher levels of chlorophyll in the transgenics demonstrated that *OsSta2* expression was positively correlated with improved salt tolerance (**Figure 5C**).

Under high-salinity stress, *Fv/Fm* values for WT plants were reduced from 0.81 to 0.66 which were 18.52% reduction. *Fv/Fm* values for transgenics plants were reduced from 0.81 to 0.70 which were 13.58% reduction and were 5% better than that of WT plants (**Figure 5D**). In contrast, under drought or low-temperature stress, *Fv*/*Fm* values were similar between the *OsSta2*-Ox and WT plants.

### Overexpression of *OsSta2* Increases Grain Yields

**FIGURE 6:**
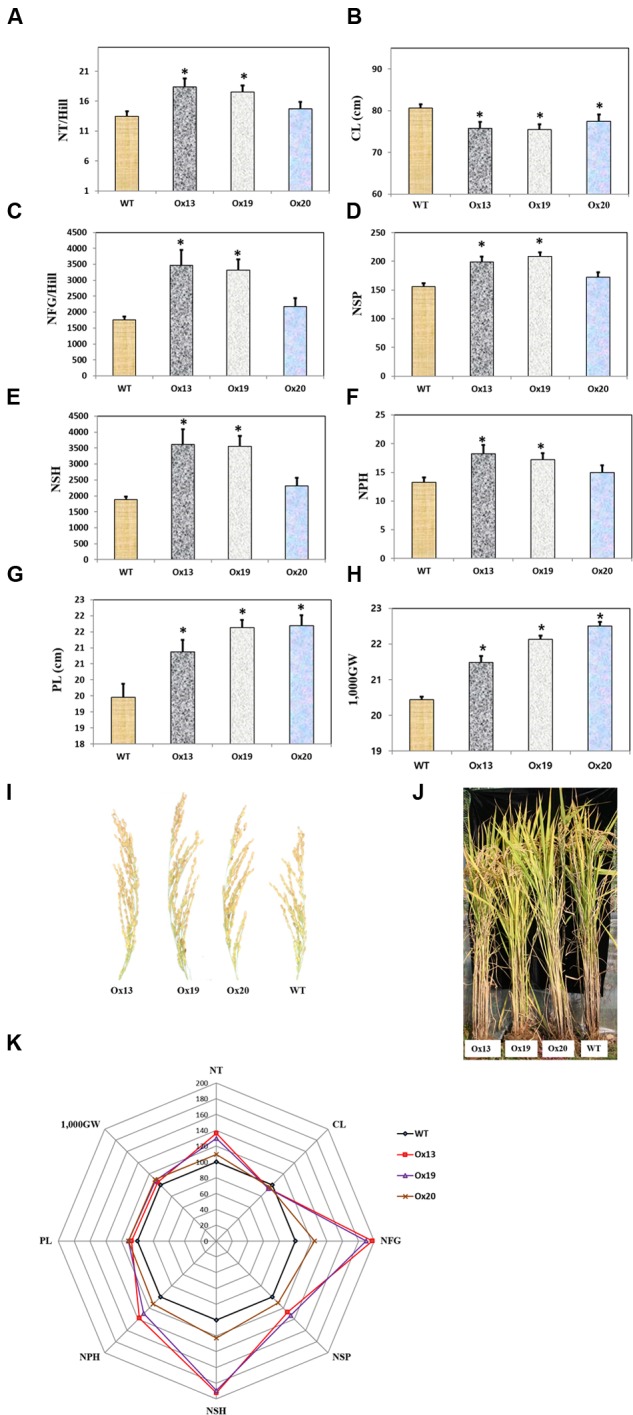
**Agronomic traits of field-grown rice plants under normal conditions. (A)** Number of tillers per hill. **(B)** Culm length. **(C)** Number of filled grains per hill. **(D)** Number of spikelets per panicle. **(E)** Number of spikelets per hill. **(F)** Number of panicles per hill. **(G)** Panicle Length. **(H)** 1,000-grain weight. **(I)** Morphology of mature rice panicle. **(J)** Morphology of mature paddy field-grown rice plant. **(K)** Spider plot of agronomic traits of three independent homozygous T5 lines of *OsSta2-*Ox and corresponding WT controls under normal conditions was drawn using Microsoft Excel software. Each data point represents percentage of mean values (*n* = 8). Mean value for WT controls was set at ‘100%’ as reference. CL, Culm length; NFG, number of filled grains; NSH, number of spikelets per hill; NSP, number of spikelets per panicle; NP, number of panicles per hill; NT, Numbers of tillers per hill; 1,000GW, 1,000-grain weight. Bars indicate standard error of 8 replicates. ^∗^, differences between Ox line and WT are significant at *P* < 0.05.

Three independent homozygous lines of *OsSta2*-Ox, together with the WT control, were grown in a paddy field. Mature transgenic plants showed semi-dwarfism but this phenotypic difference from the WT was not apparent at the four-leaf stage. Culms were 7–9% shorter from the Ox plants, i.e., 79–82 cm versus 86 cm for the WT stems (**Figure 6**). However, the Ox plants produced more tillers than the WT control, and grain yields were higher from those transgenics under normal field conditions. In particular, the grain filling rates were 17 and 23% higher for Ox13 and Ox19, respectively, than for the WT, and total grain weights were increased by 5–8% over the WT total. Filling rates did not differ significantly between Ox20 and the WT, suggesting that *OsSta2* expression was lower in that transgenic line. Nevertheless, the total grain weight was 10% higher in Ox20 than in the WT, perhaps because plants of the former type produced 8% more spikelets per panicle. Taken together, these results again showed that overexpression of *OsSta2* can improve grain yields significantly.

### *OsSta2-*Ox Transgenic Plants are Hypersensitive to ABA

**FIGURE 7:**
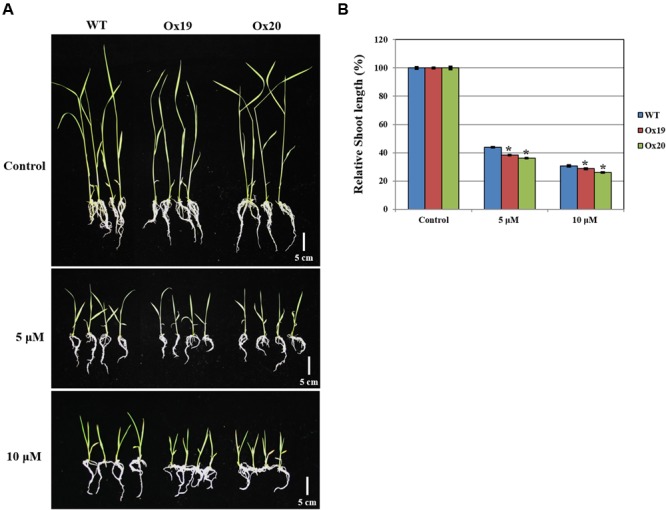
**Analysis of ABA sensitivity.** *OsSta2*-Ox rice showing ABA-sensitive phenotypes at early seedling stage. At 5 DAG, two independent Ox lines and WT control were transferred to half-strength MS media supplemented with different ABA concentrations. **(A)** Growth inhibition by ABA treatment. Photographs show representative seedlings 7 days after transfer. **(B)** Relative shoot lengths of control and Ox lines. Results are mean ± SE (*n* = 9 seedlings per experiment). Bar = 5 cm. ^∗^, differences between Ox line and WT are significant at *P* < 0.05.

Although the growth of WT shoots was repressed by ABA, this inhibitory effect was more significant in shoots from Ox plants (**Figure 7**). For example, after treatment with 5 μM ABA, relative shoot lengths from Ox plants were 61.76–63.89% shorter than those measured from plants not exposed to ABA. By comparison, the shoots from ABA-treated WT plants were 56.2% shorter than those of the WT control (untreated) plants. A similar pattern was found in response to 10 μM ABA (i.e., Ox shoots from ABA-treated plants were 71.4–74.1% shorter than those from untreated transgenics while shoots from ABA-treated WT plants were 69.5% shorter than their untreated counterparts). These results suggested that *OsSta2* is hypersensitive to ABA and is involved in its signaling pathway.

### *OsSta2*-Ox Transgenic Plants are more Tolerant than the WT to Osmotic Stress

**FIGURE 8:**
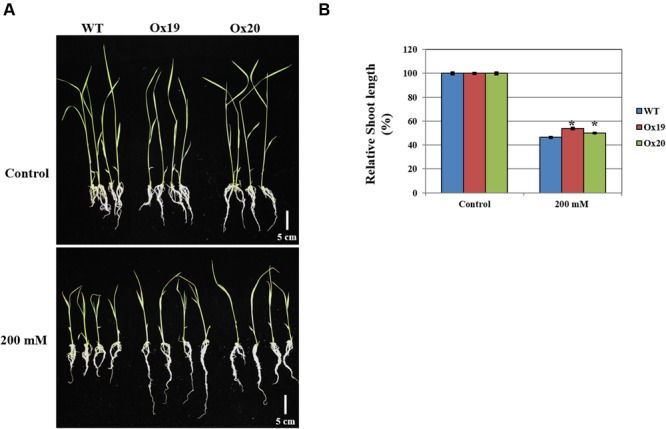
**Analysis of tolerance to osmotic stress.** *OsSta2*-Ox rice showing normal phenotypes at early seedling stage. At 5 DAG, two independent Ox lines and WT control were transferred to half-strength MS media supplemented with 200 mM mannitol. **(A)** Growth in presence of mannitol. Photographs show representative seedlings 7 days after transfer. **(B)** Relative shoot lengths of WT control and Ox lines. Results are mean ± SE (*n* = 9 seedlings per experiment). Bar = 5 cm. ^∗^, differences between Ox line and WT are significant at *P* < 0.05.

When grown in a half-strength MS medium supplemented with 200 mM mannitol, the shoots from Ox plants were 46–50% shorter than those from the untreated transgenics while shoots from mannitol-treated WT plants were 54% shorter than those from the untreated WT (**Figure 8**). This demonstrated that *OsSta2* helps confer tolerance to osmotic stress.

## Discussion

Rice is a salt-sensitive crop at the germination stage, but becomes more tolerant as plants progress from young seedlings to the vegetative stage (Heenan et al., 1988; Lutts et al., 1995). We used various assays to monitor activation or overexpression of *OsSta2* and to determine how this gene can confer enhanced salt tolerance at the seedling stage. Fujimoto et al. (2000) have reported that AtERF5 (At5g47230), acts as a functional activator of GCC box-mediated transcription. AtERF5 also plays a role as a positive regulator of JA/ethylene-mediated defenses against *Botrytis cinerea* (Moffat et al., 2012). However, no previous research confirmed its function under abiotic stresses, although a role for ERF TFs has been suggested (Nakano et al., 2006). We found here that *OsSta2* could respond to salt, drought, and ABA treatments because its promoter region contains multiple stress-related *cis*-elements, i.e., ABREs, DRE/CRT, and a MYB/MYC recognition site. Those same elements also occur in the promoter region of stress-responsive genes that are regulated by DREB, ERF, and MYB/MYC TFs, respectively (Urao et al., 1993; Baker et al., 1994; Abe et al., 1997, 2003; Guan et al., 2000; Simpson et al., 2003; Tran et al., 2004; Kaplan et al., 2006). All of these findings provide evidence of the role that *OsSta2* has in conferring salt tolerance.

Grain yield is an important parameter when investigating the effects of abiotic challenges. Overexpression of stress-related genes can alter the productivity and overall architecture of rice plants (Oh et al., 2009; Jeong et al., 2010; Xia et al., 2012; Alam et al., 2014; Yoon et al., 2016). Therefore, it was important that we examine grain yields using stable transgenic lines that did not segregate under paddy field conditions. This approach facilitated our identification of a segregating line of transgenic rice plants up to the T3 generation, even if they were homozygous for a particular transgene. To determine how yields were increased in *OsSta2*-Ox rice under normal conditions, we relocated T4 homozygous lines in 2014 and T5 homozygous lines in 2015 to the paddy field. Those lines had been pre-screened for segregation in the field from 2011 to 2013. Grain production was significantly improved in the Ox plants when compared with the WT, mainly because the former type of plant had more tillers and panicles, and its panicles were longer than those of the WT.

During its response to osmotic stress, plants utilize the ABA signaling transduction pathway to initiate the expression of defense genes (Chinnusamy et al., 2004; Singh et al., 2015). Overexpression of some stress-related genes, e.g., *OsZIP72* and *OsABI5*, results in abiotic-stress tolerance and causes the transgenic plants to be hypersensitive to exogenous ABA (Zou et al., 2008; Lu et al., 2009; Kim et al., 2014). We also found that *OsSta2*-Ox plants showed increased responsiveness to exogenous ABA, which suggested that this gene has a role in the ABA pathway during the stress response. Therefore, we proposed that *OsSta2* has a role in the ABA signaling pathway and that this response to salinity is ABA-dependent.

When plants recognize and respond to stress in an ABA-hypersensitive manner, the processes associated with physiological processes may retard growth because necessary resources are instead being directed toward mechanisms for protection. This can occur even under normal environmental conditions because higher levels of transcripts for genes related to abiotic-stress tolerance can inhibit plant development, especially when such genes are constitutively over-expressed. This is particularly true for genes associated with ABA signaling because that phytohormone has important regulatory roles (Sreenivasulu et al., 2012). For example, rice plants that constitutively over-express DREB1A grow more slowly under standard conditions (Kasuga et al., 1999; Nakashima et al., 2007). This might explain why our *OsSta2*-Ox plants showed slight retardation when grown to maturity in the paddy field. However, no such inhibition was noted when young *OsSta2*-Ox seedlings were grown either in soil boxes in a chamber or on plates containing a half-strength MS medium. To partially overcome this problem when conducting experiments, researchers utilize promoters that are stress-inducible, such as *OsDhn1, rd29A*, and *OsPOX1* (Kasuga et al., 1999; Wang et al., 2005; Kim S.H. et al., 2012; Lee et al., 2013; Kumar et al., 2014).

As with salt stress, *OsSta2*-Ox plants were also more tolerant to osmotic stress, maintaining a much healthier growth pattern (as reflected in shoot length parameters) than the WT seedlings in response to mannitol treatment. Similar results have been described previously (Zou et al., 2012; Kumar et al., 2013; Kim et al., 2014; You et al., 2014; Singh et al., 2015). Hence, we can conclude that *OsSta2*-Ox plants exhibit ABA-dependent salt tolerance via osmotic stress signaling.

The extent to which Ox lines are salt-tolerant also depends on the level of *OsSta2* expression and its involvement in the tolerance pathway (Ashraf, 2009). Because salinity-promoted oxidative stress is peripheral to ionic and osmotic stresses, it might not be possible to achieve adequate salt tolerance through the manipulation of *OsSta2* alone but it might entail strong interactions with other stress-related genes (Ashraf, 2009; Kumar et al., 2013). Therefore, further exploration of such genetic inter-relationships is necessary if we are to produce crop plants that are more tolerant to environmental challenges.

## Conclusion

We have demonstrated that overexpression of *OsSta2* enhances the tolerance of transgenic rice plants to salt and osmotic stresses. This is manifested by an increase in tiller numbers and grain yields. However, additional analyses of gene expression and how they finely regulate plant processes are required in the future.

## Author Contributions

MK and S-RK design experiments and wrote manuscript. MK performed all the experiments. All the authors approved final manuscript.

## Conflict of Interest Statement

The authors declare that the research was conducted in the absence of any commercial or financial relationships that could be construed as a potential conflict of interest.

## Footnotes

1 http://signal.salk.edu/cgi-bin/RiceGE5

2 http://www.generunner.net/

3 http://www.ncbi.nlm.nih.gov/tools/primer-blast/

4 http://tenor.dna.affrc.go.jp/

5 https://dbarchive.biosciencedbc.jp/en/kome/desc.html

## Abbreviations

ABA: abscisic acid
ATL: activation tagging line
DAG: days after germination
LSD: least significant difference
MS: Murashige and Skoog
