# Supplementary material for: Ectopic Expression of OsSta2 Enhances Salt Stress Tolerance in Rice
Source: Front Plant Sci. 2017 Mar 10;8:316. doi: 10.3389/fpls.2017.00316 (PMC5344931; doi:10.3389/fpls.2017.00316)
Supplement: Supplementary file 1 [file Data_Sheet_1.docx]

Supplementary data

**Ectopic expression of OsSta2 enhances salt stress tolerance in rice**

**Manu Kumar^1^, Juyoung Choi^1^, Gynheung An^2^, and Seong-Ryong Kim^1,^***

*^1^Department of Life Science, Sogang University, Seoul 121-742, Korea, ^2^Department of Plant Molecular Systems Biotechnology, Kyung Hee University, Yongin 446-701, Korea*

*Corresponding Author: Seong-Ryong Kim, PhD

Department of Life Science, Sogang University,

Seoul 121-742, Korea

Tel: +82-02-705-8456, FAX: +82-2-704-3601

E-mail: [sungkim@sogang.ac.kr](mailto:sungkim@sogang.ac.kr)

**Figure S1. Distribution of major stress related cis-acting elements in the promoter region of OsSta2.**

**Figure S2. Construction of transgenic rice overexpressing *OsSta2.*** Genomic-DNA PCR of 21 independently generated *OsSta2*-Ox transgenic rice to check the insert by using forward primer from *ubiquitin* promoter region (Pubi-F) and reverse primers from *OsSta2* gene (OsSta2-R).

**Figure S3. Transgenic rice overexpressing *OsSta2* exhibits enhanced salt and tolerance at the vegetative stage. (A**) Visual phenotype of salt tolerance response of transgenic plants [sense *OsSta2*] and WT after 250 mM salt treatment for 72 h of 8 DAG rice followed 6 d recovery. **(B)** Fresh weight after recovery. **(C)** Dry weight after drying for 2 days. Asterisks (*) value shows significant difference between OX line, and WT (*t-* test, *P*<0.05).

**Figure S4. RT-PCR to amplify the fragment from AP25 domain. (A**) Schematic diagram of two transcript **(B)** RT-PCR using different primers with in the transcript.

**Figure S5. Stages of homozygosity (T0-T5) along with the type of experiments performed.**

**FigureS6. Southern blot of *OsSta2*.** Genomic DNA from *OsSta2*-Ox lines were digested with *EcoR*1 (R1), B*amH*1 (Bm) and *Hind*III (H3). The blot was hybridized with *OsSta2* probe. The positions and sizes (in kbp) are indicated on the right side.

**Table S1. Potential stress-related *cis*-acting elements in the promoters of *OsSta2*.**

| **Cis-acting**  **elements** | **Sequence** | **Copy**  **no.** | **Function** | **References** |
| --- | --- | --- | --- | --- |
| W-box | TTGAC, TGACT,  TGAC, TGACY,  TTTGACY | 39 | Involved in activation of genes  involved in response to wounding  and defense | Chen et al., 2002,  Xie et al., 2005 |
| GT 1 | GRWAAW, GGTTAA,  GAAAAA, | 29 | Influences the level of SA-inducible gene expression | Buchel et al., 1999 |
| MYB | WAACCA, YAACKG,  CNGTTR, AACGG,  CCWACC, GGATA,  TAACTG, | 20 | Involved in regulation of drought  inducible gene expression | Urao et al., 1993,  Abe et al., 1997 |
| MYC | CATGTG, CACATG,  CANNTG | 20 | Involved in drought- and ABA-  regulated gene expression | Tran et al., 2004,  Abe et al., 2003 |
| GATA-box | GATA | 15 | chlorophyll a/b binding protein | Reyes et al., 2004 |
| ABRE | ACGTG,  MACGYGB,  CCACGTGG | 13 | ABRE-like sequence required for  etiolation-induced expression of erd1 (early responsive to dehydration) | Simpson et al., 2003,  Kaplan et al., 2006,  Guan et al., 2000 |
| Erd1 | ACGT | 12 | Required for early response to  dehydration | Simpson et al., 2003 |
| CURECORECR | GTAC | 8 | Involved in oxygen-response | Quinn et al., 2002 |
| BIHD1OS | TGTCA | 8 | Involved in disease resistance  responses | Luo et al., 2005 |
| DPBFCOREDCDC3 | ACACNNG | 4 | Involved in ABA response | Finkelstein et al., 2000, Kim et al., 1997, Lopez-Molina et al., 2000 |
| CGCG box | VCGCGB | 2 | Involved in ethylene signaling,  abscisic acid signaling, and light  signal perception | Yang et al., 2002 |
| RAV | CAACA | 1 | RAV1 protein recognition sequence | Kagaya et al., 1999 |
| GCC-box | GCCGCC | 1 | Ethylene-responsive element | Baker et al., 1994 |
| CBF | RYCGAC | 1 | Dehydration-responsive element  (DRE) binding proteins (DREBs) | Xue GP., 2002 |
| ERE | AWTTCAAA | 1 | Ethylene responsive element | Itzhaki et al., 1994,  Montgomery et al., 1993 |

**Table S2. Primers used in this study**

|  | | |
| --- | --- | --- |
| **Name** | **Sequence (5’- 3’)** | **Purpose** |
| P1 (*OsSta2-F*) | AGATGCAATTGCGCGAAC | RT-PCR, qRT-PCR |
| P2 (*OsSta2-R*) | CACCGTTGAGGTTGTCGTTG | RT-PCR |
| P3 | AGATGCAATTGCGCGAAC | RT-PCR |
| P4 | TCGGTCTTGACGGATTTGGA | RT-PCR, qRT-PCR |
| P5 | AGATCCGCGACCCCAAGAAG | RT-PCR |
| P6 | AGTCCCCTCTCCTCTCTCGT | RT-PCR |
| P7 | TACGTGGACATGGCGAATGA | RT-PCR |
| P8 | CGGAGGTCGCCGTAAACTAA | RT-PCR |
| P9 | GCTCGGCACGTACGACAC | RT-PCR |
| P10 | TTTACAATGTTATTATCACCCATCC | RT-PCR |
| *Pubi-F* | TTGATATACTTGGATGATGGCATA | gD-PCR |
| *Tnos-R* | GCGGGACTCTAATCATAAAAACC | gD-PCR |
| *RAc1-F* | CGCAGTCCAAGAGGGGTATC | RT-PCR |
| *RAc1-R* | TCCCTCACAATTTCCCGCTC | RT-PCR |
| *RAc1-qF* | GACTCTGGTGATGGTGTCAGCCACAC | qRT-PCR |
| *RAc1-qR* | CGCACTTCATGATGGAGTTGTAT | qRT-PCR |
| L0.5 | TTGGGGATCCTCTAGAGTCGAG | 1^st^ IPCR |
| cla1-LB-R1 | TGGCTCCGATCTTAAGAAAAGAACAGT | 1^st^ IPCR |
| L1.5S | AGTACATTAAAAACGTCCGC | 2^nd^ IPCR |
| cla1-LB-R2 | GCCTAATCAACATCTCTGCTATCTGC | 2^nd^ IPCR |
| *LOC_Os02g43830* For. | GATGTGGTCACGGTGGAACT | RT-PCR |
| *LOC_Os02g43830* Rev. | ACCACTGATGACACACAACCT | RT-PCR |
