# Supplementary figures and images for: Ectopic Expression of OsSta2 Enhances Salt Stress Tolerance in Rice
Source: Front Plant Sci. 2017 Mar 10;8:316. doi: 10.3389/fpls.2017.00316 (PMC5344931; doi:10.3389/fpls.2017.00316)

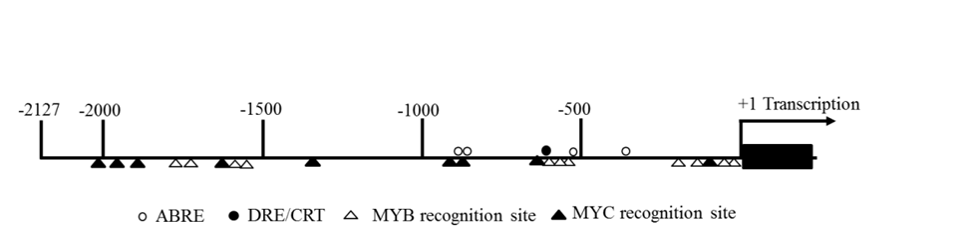

Supplement: Supplementary file 2 [file Image_1.tif]

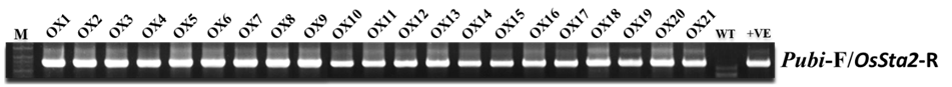

Supplement: Supplementary file 3 [file Image_2.tif]

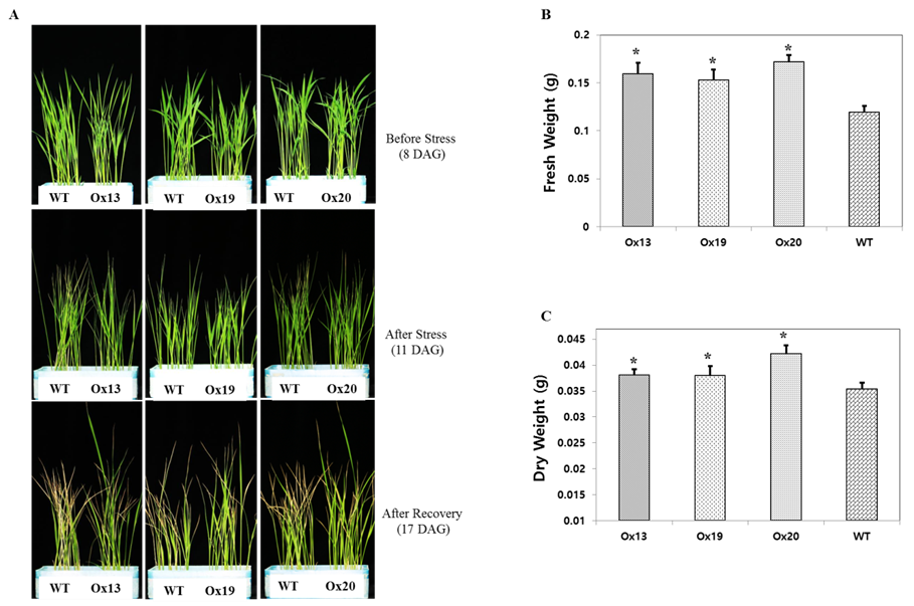

Supplement: Supplementary file 4 [file Image_3.tif]

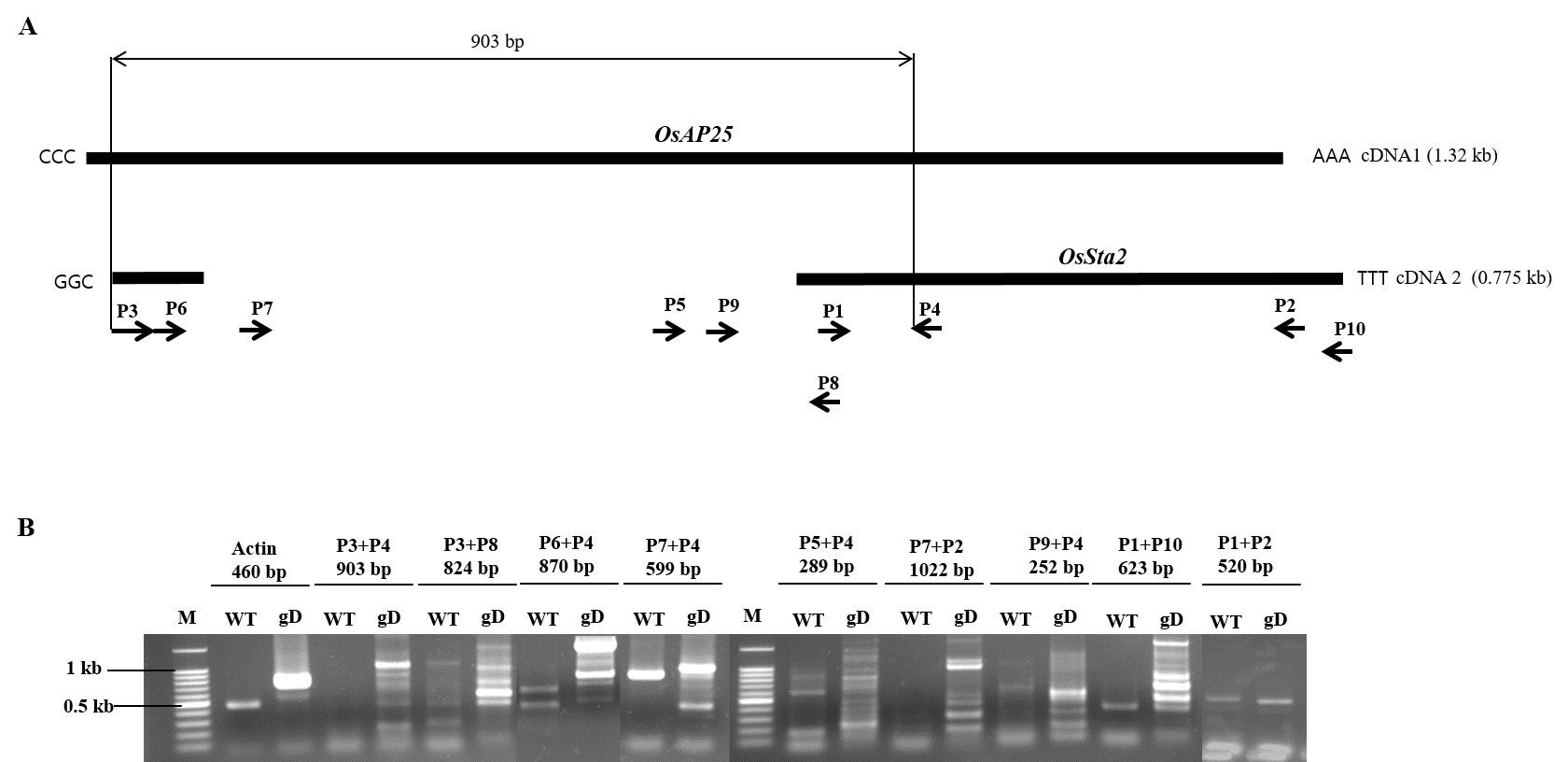

Supplement: Supplementary file 5 [file Image_4.tif]

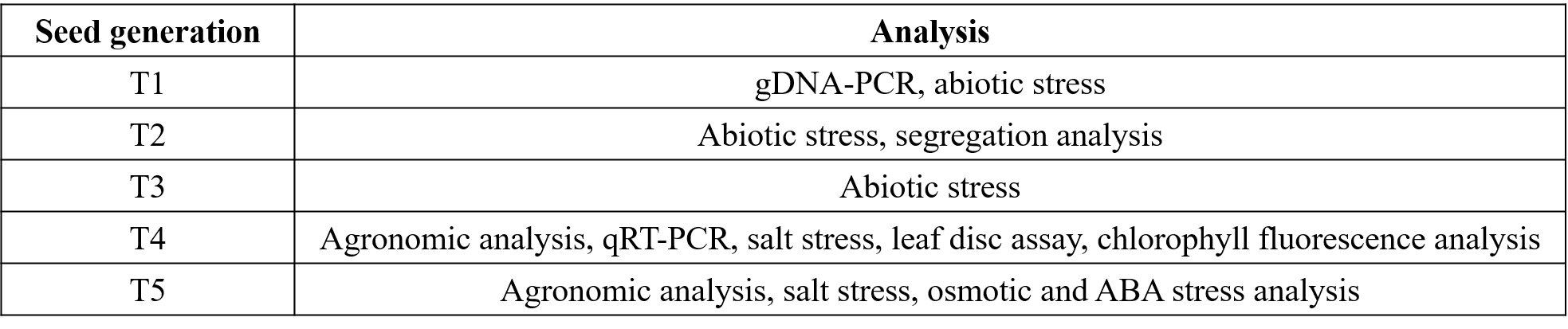

Supplement: Supplementary file 6 [file Image_5.tif]

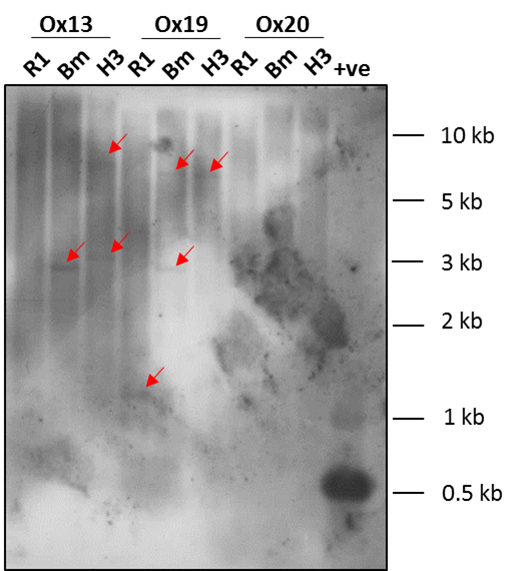

Supplement: Supplementary file 7 [file Image_6.tif]
